# Nurses’ competence in recognition and management of delirium in older patients: development and piloting of a self-assessment tool

**DOI:** 10.1186/s12877-022-03573-8

**Published:** 2022-11-19

**Authors:** Jonas Hoch, Jürgen M. Bauer, Martin Bizer, Christine Arnold, Petra Benzinger

**Affiliations:** 1 Center for Geriatric Medicine, Heidelberg University Hospital, AGAPLESION Bethanien Hospital Heidelberg, Rohrbacher Strasse 149, 69126 Heidelberg, Germany; 2 Department of General Practice and Health Services Research, Heidelberg University Hospital, Im Neuenheimer Feld 130.3, 69120 Heidelberg, Germany; 3 Network Aging Research (NAR), Heidelberg University, Bergheimer Strasse 20, 69115 Heidelberg, Germany; 4 Department of Internal Medicine, Heidelberg University Hospital, Im Neuenheimer Feld 672, 69120 Heidelberg, Germany; 5 Institute of Health and Generations, University of Applied Sciences Kempten, Bahnhofstrasse 61, 87435 Kempten, Germany

**Keywords:** Delirium, Delirium recognition, Delirium management, Clinical reasoning, Educational intervention, Vignette-based questionnaire, Nursing, Geriatric, Older adult

## Abstract

**Background:**

Delirium is a common condition in elderly inpatients. Health care professionals play a crucial role in recognizing delirium, initiating preventive measures and implementing a multicomponent treatment strategy. Yet, delirium often goes unrecognized in clinical routine. Nurses take an important role in preventing and managing delirium. This study assesses clinical reasoning of nurses using case vignettes to explore their competences in recognizing, preventing and managing delirium.

**Methods:**

The study was conducted as an online survey. The questionnaire was based on five case vignettes presenting cases of acutely ill older patients with different subtypes of delirium or diseases with overlapping symptoms. In a first step, case vignettes were developed and validated through a multidisciplinary expert panel. Scoring of response options were summed up to a Geriatric Delirium Competence Questionnaire (GDCQ) score including recognition and management tasks The questionnaire was made available online. Descriptive analyses and group comparisons explores differences between nurses from different settings. Factors explaining variance in participants’ score were evaluated using correlations and linear regression models.

**Results:**

The questionnaire demonstrated good content validity and high reliability (kappa = 0.79). The final sample consisted of 115 nurses. Five hundred seventy-five case vignettes with an accuracy of 0.71 for the correct recognition of delirium presence or absence were solved. Nurses recognized delirium best in cases describing hyperactive delirium (79%) while hypoactive delirium was recognized least (44%). Nurses from geriatric and internal medicine departments had significantly higher GDCQ-score than the other subgroups. Management tasks were correctly identified by most participants.

**Conclusions:**

Overall, nurses’ competence regarding hypoactive delirium should be strengthened. The online questionnaire might facilitate targeting training opportunities to nurses’ competence.

**Supplementary Information:**

The online version contains supplementary material available at 10.1186/s12877-022-03573-8.

## Background

Delirium is a neuropsychiatric syndrome characterized by acute disturbance of attention, consciousness, cognitive function or perception with a fluctuating course [1, 2]. Symptoms might present as hypoactive, hyperactive, or mixed motoric subtypes. Delirium occurs across all healthcare settings but is most common in acutely hospitalized patients. Older age is a strong predisposing factor in hospitalized patients resulting in a higher chance to suffer a delirium [3]. At the same time, multiple risk factors might trigger delirium onset like acute illness, trauma, surgery, and medications. The prevalence of delirium is variable across various departments and might be as high as > 20% in intensive care units and in emergency departments [1, 4].

Although symptoms resolve within days in most patients, cognitive deficits might persist for months. Delirium is associated with adverse outcomes such as functional decline, institutionalization, dementia, and mortality [5–8]. Often, delirium is distressing for patients as well as their caregivers [9]. While treated in hospital, patients with delirium need more attention from nursing staff which leads to a higher workload [10, 11]. Furthermore, patients suffering from delirium have a longer length of stay resulting in higher costs per case [7, 12].

Delirium is a clinical bedside diagnosis based on recognition of its characteristic features by healthcare professionals [13]. There is sufficient evidence that a multicomponent nonpharmacological approach can effectively prevent the onset of delirium and reduce symptom duration [14, 15]. For successful implementation and maintaining of a multi-dimensional diagnostic and therapeutic approach, interprofessional collaboration of physicians, nurses, therapists, as well as family members and trained volunteers is imperative [16]. Nurses play a key role in prevention and detection of delirium [17]. They spend more time in direct contact with patients than any other healthcare profession. Their attitudes and knowledge are critical to delirium recognition and management [18]. In Germany, there is currently only a limited focus on delirium in the national nursing education curriculum. However, gaps in nurses’ knowledge and understanding of delirium have already been demonstrated elsewhere [19, 20]. In a response to this knowledge gap, an interdisciplinary statement of scientific societies specifically addressed the need for better training of healthcare professionals, and nurses in particular [16].

To make an impact on care, training of nurses should increase their clinical competences and clinical reasoning skills [21]. Clinical reasoning describes the process health care professionals go through in their daily routine to successfully solve simple to complex patient encounters. Clinical reasoning consists of clinical judgement and clinical decision making. While clinical judgment involves the process of recognizing what is wrong with the patient, clinical decision making includes adoption of preventive measures and the management of clinical problems [22]. Single choice questionnaires often do not sufficiently represent this complex process of clinical reasoning. To this end, case vignettes are established for training of medical students and physicians since they are more suitable to assess clinical judgement and decision making. Up to now, case vignettes are less frequently used for training of nursing students and nurses [23]. In the context of delirium, surveys conducted in Canada and the United States used case vignettes to assess nurses’ recognition of delirium [24–28]. So far, very few surveys focused on using case vignettes to assess the whole clinical reasoning process by nurses including recognition as well as management of delirium in this detail.

The aim of this study was to develop and to pilot a self-assessment instrument for nurses to evaluate their clinical reasoning skills in recognition and management of delirium in geriatric patients using case vignettes.

## Methods

### Study design and study population

The online version of the questionnaire was developed using LimeSurvey (Version 3.22.1 + 200,129, hosted by Heidelberg University). After ethical approval, participants were recruited between August 2021 and October 2021 through personal communication, professional organizations, and providers of continuing training. Respondents could access the survey via a link or a QR code. Due to the anonymous design, the survey was open to other health care professionals. Inclusion criteria were (1) a nursing degree and (2) current employment at a hospital, in post-acute or long-term facilities. Respondents not meeting the inclusion criteria were excluded from further analyses.

### Development of case vignettes and questionnaire

**Fig. 1:**
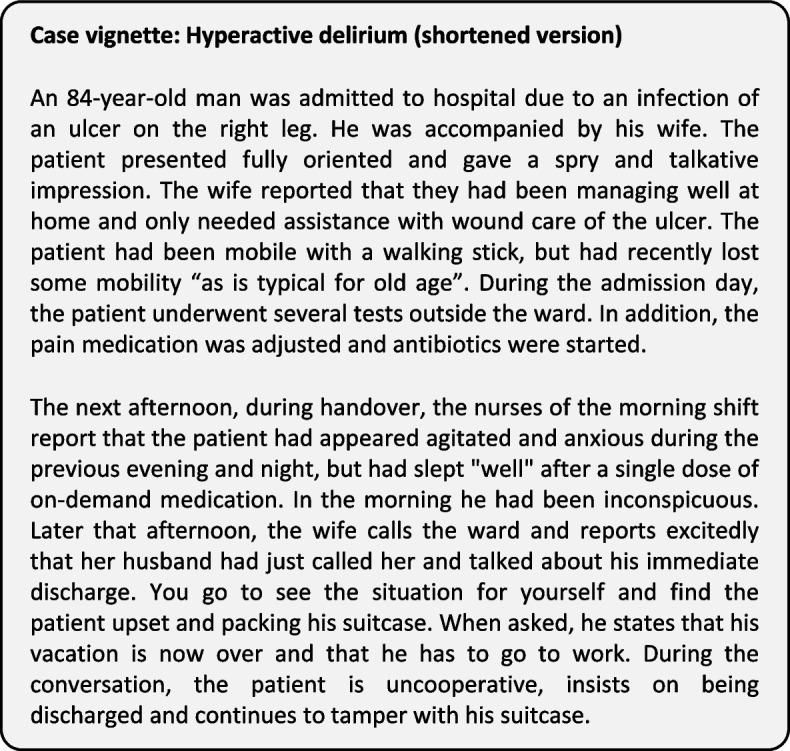
Abridged example of the case vignette with hyperactive delirium (short version)

The questionnaire was designed in order to assess delirium competence using five case vignettes describing scenarios in a general hospital characterizing patients suffering from different subtypes of delirium (hypoactive, hyperactive, and hyperactive superimposed on dementia) or diseases with overlapping symptoms (dementia, depression). Two authors (JH, MB) developed the case vignettes through an iterative process based on previously published vignette studies [24, 26, 29], review of literature and clinical relevance as judged by the authors. Careful consideration was given to the content of the vignettes so that they closely related to real clinical scenarios and included information that would facilitate delirium recognition. All vignettes presenting delirious patients described clinical signs as covered by the Confusion Assessment Method (CAM), a well-established instrument for detection of delirium [30]. Clinical signs were defined as acute onset, fluctuating course in mental status, and inattention with additional symptoms of disorganized thinking or altered levels of consciousness. A shortened example of a case vignette is presented in Fig. 1, all case vignettes are provided as Supplementary Material.

All case vignettes included questions about recognition of delirium including delirium subtypes, prevention and further management tasks. The questions were primarily based on selected response formats and included true/false, single-choice, short menu formats and multiple response questions [31]. The vignettes were reviewed by a geriatrician (PB).

For content validity, the questionnaire was reviewed by two psychiatrists, one physician and two nurses with a master’s degree. All had experience in clinical research, geriatrics, and delirium.

Feasibility and comprehension were tested by three nurses with a low level of self-reported experience in delirium management. They needed 25 to 35 minutes for completion of the questionnaire. Consequently, the questionnaire was shortened to reduce administration time.

To measure reliability of agreement, the survey was completed by five nurses with a master’s degree. Fleiss’ kappa was used for statistical analyses. They demonstrated 100% inter-rater agreement with the correct identification of delirium presence or absence for each case and an overall kappa of 0.79. Results between 0.61 and 0.80 can be considered as substantial agreement, results between 0.81 and 1.0 as almost perfect [32]. No further adaptation of the case vignettes was warranted.

Nurses who participated in the review process were excluded from the pilot study.

### Measurement scales and independent variables

For further statistical analysis, questions of the case vignettes were aggregated to constitute a Geriatric Delirium Competence Questionnaire (GDCQ-score) with a score ranging from zero to 55. The score consisted of questions related to clinical judgement as well as clinical decision making (see Supplementary Material).

After completion of the case vignettes, participants were grouped by their current work environment (‘geriatric and internal medicine departments’, ‘other acute hospital departments’, and ‘post-acute and long-term care facilities’). The subgroup ‘other acute hospital departments’ consisted of nurses working on any inpatient ward including intensive care units (ICU) and psychiatric wards.). Furthermore, they were asked about their previous delirium training (accumulated hours in total, training within the previous 12 months), work experience with delirious patients and satisfaction with delirium management at their current work place using a Likert-scale (1–5, higher = more frequent / higher satisfaction). Participants were asked to self-assess their knowledge on delirium before starting and after completion of the case vignettes using a Likert-scale (1–5, higher = more knowledge). The independent variable, frequent treatment of delirious patients in daily routine, was dichotomized (very often, often = 1, less = 0).

### Statistical analysis

Statistical analyses were performed using the R Foundation for Statistical Computing 4.1.0. Descriptive variables were described by means and standard deviation, median and interquartile range, or percent. Differences between subgroups were tested by using the non-parametric Kruskal-Wallis-Test, which is distributed as a chi-square. Group comparisons of dichotomous variables and the GDCQ-score were performed using two-sided Welch T-test [33]. Five-point Likert-scales were treated as continuous variables in correlation analyses and further regression models [34, 35]. Pearson’s correlation was used to test for correlations between GDCQ-score and independent variables. Univariate linear regression analyses were performed with GDCQ-score as dependent variable. Level of significance was set at *p* < 0.05 (two-tailed) for all analyses.

## Results

### Sample characteristics

Between August and October 2021, the survey was started 248 times of which 51% times respondents (*n* = 126) completed the questionnaire. Mean completion time of the survey was 22.2 minutes (SD 9.6 minutes). Case vignettes presented in this questionnaire were rated as ‘very good’ or ‘good’ by 88% of participants. Respondents who identified themselves as nurses were included for further analyses (*n* = 115). The average work experience of participants was 19.6 years. Fifty-two participants worked in geriatric or internal medicine departments, and 33% of participating nurses had a specialist nursing qualification in geriatric medicine. Of nurses working in geriatric and internal medicine departments, 61% reported frequent treatment of delirious patients in their departments while nurses working in other departments or facilities reported frequent treatment of delirious patients significantly less often (‘other acute care’ departments 40%, ‘post-acute and long-term care facilities’ 8%). Nearly every other nurse working in geriatric and internal medicine department reported participation in delirium training within the previous 12 months (Table 1).

**Table 1:** Characteristics of the sample according to hospital departments and care facilities

	total sample ***n*** = 115	geriatric and internal medicine departments ***n*** = 50	other acute hospital departments ***n*** = 52	post-acute and long-term care facilities ***n*** = 13	***p***-value^**1**^
gender (female)^a^	93 (80%)	43 (86%)	38 (72%)	12 (92%)	0.14
work experience (years)^b^	19.6 (11.3)	22.7 (11.0)	17.8 (10.6)	14.5 (12.4)	0.02
geriatric nursing qualification^a^	38 (33%)	26 (52%)	8 (15%)	4 (31%)	< 0.001
delirium training within the previous 12 months^a^	38 (33%)	23 (46%)	13 (25%)	2 (15%)	0.03
accumulated delirium training (total hours)^b^	11.0 (14.0)	13.4 (17.2)	10.0 (11.1)	4.2 (6.6)	0.21
frequent treatment of delirious patients in daily routine^a^	53 (46%)	31 (62%)	21 (40%)	1 (8%)	< 0.01

^a^N(%)

^b^Mean (SD)

^1^Kruskal-Wallis Test

### Recognition of delirium

Overall, participants completed 575 case vignettes with an accuracy of 0.71 for the correct recognition of delirium presence or absence. The correct subtype of delirium was recognized by 48% of participating nurses. Nurses working in geriatric and internal medicine departments identified hyperactive delirium significantly better than nurses from post-acute and long-term care facilities (*p* < 0.01). There were no statistically significant differences between subgroups for the recognition of delirium in all other case vignettes (Table 2).

**Table 2:** Clinical judgement: Correct recognition of delirium presence or absence and correct diagnoses of the cases

Case vignette	sample^a^***n*** = 115	geriatric and internal medicine departments^a^***n*** = 50	other acute hospital departments^a^***n*** = 52	post-acute and long-term care facilities^a^***n*** = 13	***p***-value^**2**^
	**recognition of delirium /** correct delirium subtype	**recognition of delirium /** correct delirium subtype	**recognition of delirium /** correct delirium subtype	**recognition of delirium /** correct delirium subtype	
hyperactive delirium	**91 (79%)** / 62 (54%)	**44 (88%)** / 29 (58%)	**41 (79%)** / 28 (54%)	**6 (46%)** / 5 (38%)	**< 0.01** / 0.46
delirium superimposed on dementia	**70 (61%)** / 55 (48%)	**31 (62%)** / 26 (52%)	**31 (60%)** / 24 (46%)	**8 (62%)** / 5 (38%)	**0.97** / 0.65
hypoactive delirium	**51 (44%)** / 50 (43%)	**23 (46%)** / 23 (46%)	**25 (48%)** / 24 (46%)	**3 (23%)** / 3 (23%)	**0.26** / 0.29
	**recognition of delirium absence /** correct diagnoses	**recognition of delirium absence /** correct diagnoses	**recognition of delirium absence /** correct diagnoses	**recognition of delirium absence /** correct diagnoses	
depression	**110 (96%)** / 80 (70%)	**47 (94%)** / 38 (76%)	**51 (98%)** / 38 (73%)	**12 (92%)** / 4 (31%)	**0.50** / 0.24
dementia	**85 (74%)** / 65 (57%)	**37 (74%)** / 27 (54%)	**36 (69%)** / 30 (58%)	**12 (92%)** / 8 (62%)	**0.24** / 0.87

^a^n (%)

^2^Kruskal-Wallis Test

For recognition of delirium, most participants used clinical signs (81%) and information provided by relatives (71%). Respondents reported to use validated assessment tools including Delirium Observation Screening (DOS) (55%), Nursing Delirium Screening Scale (NuDesc) (47%), and Confusion Assessment Method (CAM) (44%). Use of no validated method to detect delirium was reported by 11% of participants.

### Management tasks

Overall, most participants were able to differentiate whether suggested measures were appropriate. Nurses working in geriatric and internal medicine departments scored higher than the other subgroups and scored significantly higher than participants from non-acute care settings in all four items although differences reached statistical significance for recognition of risk factors and initiation of preventive measures only (Table 3).

**Table 3:** Clinical decision making: Management of delirium

management tasks (Score with zero to a maximum of 10 points per task)	sample^a^***n*** = 115	geriatric and internal medicine department^a^***n*** = 50	other acute hospital department^a^***n*** = 52	post-acute and long-term care facilities^a^***n*** = 13	***p***-value^**2**^
Task 1: treatment of delirium	7.7 (1.1)	7.9 (0.9)	7.6 (1.1)	7.2 (1.5)	0.14
Task 2: recognition of risk factors and initiate preventive measures	8.8 (1.0)	9.2 (0.9)	8.7 (0.9)	7.8 (1.1)	< 0.001
Task 3: prevention for patients with cognitive impairment	9.0 (1.0)	9.3 (0.8)	8.9 (1.1)	8.4 (1.3)	0.03
Task 4: interdisciplinary communication	7.8 (2.1)	8.4 (1.6)	7.6 (2.3)	6.3 (2.3)	0.01

^a^Mean (SD)

^2^Kruskal-Wallis Test

### GDCQ-score, correlations, and linear regression models

The mean score of the sample was 42.62 (SD = 4.86) out of a maximum of 55 points. Subgroups analyses demonstrated that nurses working in geriatric and internal medicine departments scored a mean of 44.34 (SD = 4.01). Participants from other acute hospital departments scored an average of 42.17 (SD = 4.98) and participants from post-acute and long-term facilities scored a mean of 37.77 (SD = 3.81). Difference between subgroups was significant (*p* < 0.01). Further post-hoc analyses by the Wilcoxon rank sum test with continuity correction by Holm showed a significant difference between all three subgroups (*p* < 0.05).

There were significant correlations with small effect sizes between GDCQ-score and some independent variables. While overall work experience shown no significant correlation, frequent care of delirious patients in daily routine and the subjective self-assessment after survey did (Table 4).

**Table 4:** Univariate linear regression analyses for the independent variables and the Geriatric Delirium Competence Questionnaire score

independent variables	Univariable regression analyses			
	B	Standard Error	Beta (β)	***p***-value
gender (female = 1)	−0.64	1.16	−0.05	0.58
work experience (years)	0.02	0.04	0.05	0.58
frequent care of delirious patients in daily routine	2.67	0.88	0.28	< 0.01^**^
delirium training within the previous 12 months	2.3	0.95	0.23	0.02^*^
accumulated delirium training (total hours)	0.08	0.04	0.22	0.03^*^
subjective self-assessment before survey	1.45	0.4	0.33	< 0.001^***^
subjective self-assessment after survey	2.7	0.44	0.50	< 0.001^***^

^*^p-value < 0.05

^**^p-value < 0.01

^***^p-value < 0.001

## Discussion

This pilot study describes the development and piloting of a questionnaire to self-assess competence in recognition and management of delirium in older patients by nurses. Case vignettes offer the opportunity to assess nurses’ clinical skills by reflecting realistic scenarios. Our results demonstrate feasibility of the questionnaire in a German setting and allow insights into delirium competence of nurses in Germany. To our knowledge, this study is the first to assess the abilities of nurses to recognize and manage delirium using case vignettes.

Overall, delirium was detected by most nurses participating in the pilot study. In our sample, nurses were better in recognizing the absence of signs of delirium than the presence of such signs. This finding is in line with previous studies [26, 36, 37]. In a study with home care nurses, 93% of participants recognized the absence of signs of delirium in a case vignette describing depression [26]. Other studies using case vignettes describing dementia without delirium reported correct recognition of absence of delirium by 68 and 83% of participating nurses [24, 26]. Where there is uncertainty on the nature of cognitive alterations, dementia might appear to be a more obvious choice to many nurses as compared to delirium.

In the present study, presence of delirium was best recognized in a case vignette describing hyperactive delirium. Higher detection rates for hyperactive delirium as compared to other subtypes are in line with previous findings [24, 26]. Yet, hypoactive delirium is more common than hyperactive delirium in inpatient settings [38, 39]. It is associated with higher mortality and worse outcome as compared to other types of delirium [40]. One reason for poor clinical outcome of patients with hypoactive delirium might be the lower detection rates in clinical routine. During a busy shift, delirium might remain unrecognized in patients not seeking attention from nursing staff [41]. Delirium superimposed on dementia also seems to be challenging to evaluate for the participants of our study. This seems to reflect current clinical practice [42]. Low detection rates of hypoactive delirium and delirium superimposed on dementia in case vignettes, as seen in our study and previous studies, point towards gaps in nurses’ knowledge of delirium and suggest better training of health care professionals on delirium [16].

Among participants of the pilot study, nurses from post-acute and long-term facilities tended to recognize delirium less often than nurses from other settings and achieved the lowest overall GDCQ-score. These findings may in part be attributed to the content of the case vignettes. The situations described do not reflect scenarios of post-acute or long-term care settings and experiences of staff with delirious persons in these settings might be distinct from what was presented in the case vignettes. Yet, rates of correct diagnosis of delirium in this study is comparable to a larger study involving more than 500 staff members of various long-term care facilities in the United States [25]. In light of the substantial prevalence of delirium among nursing home residents, future research and efforts on delirium management should include nurses in non-acute health care settings [43–45].

Development of case vignettes should follow a robust methodology [29]. Professionals with different backgrounds were involved and pre-testing demonstrated high agreement of scoring between experts. Construct validity of the case vignettes developed was supported by univariate regression analyses. Frequent exposure to delirious patients and participation in delirium training were positively associated with higher scores indicating higher competence, while years of work experience did not explain variance of the overall score. These findings are supported by the findings of a study enrolling community health care nurses [46]. It is plausible that daily routine care for such patients and training have a strong impact on nurses’ delirium competence.

### Strengths

This study is the first in Germany using clinical case vignettes to assess nurses’ competence rather than knowledge of delirium [47]. So far, case vignettes focusing on delirium have been used to assess nurses’ ability to recognize delirium in various nursing settings [25–27, 29]. In this study, based on previous case vignettes, we developed with the help of a multi-professional team a novel questionnaire for nurses that assesses not only recognition but also management of delirium. Unlike previous studies, case vignettes in this questionnaire combined multiple-choice, multiple-response questions as well as short menu lists in order to reduce cueing. It is well suited to the German health care setting and represents situation encounters well known to nurses.

### Limitations

There are several limitations that need to be considered. First, case vignettes are developed to reflect realistic scenarios but in cases of delirium one has to acknowledge that signs of delirium often fluctuate over the course of the day, making detection of delirium even more challenging. For methodological reasons, it remains unclear how well scores obtained in the newly developed questionnaire reflect clinical reasoning in practice. Second, case vignettes developed in this questionnaire were describing older patients admitted to non-intensive care wards. They do not cover delirious patients on intensive care units, nor do they describe older patients cared for in post-acute or long-term care facilities. While there was a sufficient number of nurses from geriatric and general medical wards, the limited number of participants from other acute care departments did not allow for further exploration. A larger sample is needed to draw more generalizable conclusions. Third, we recruited a convenience sample for piloting the questionnaire. It is very likely that nurses with a particular interest in the topic visited the online site of the questionnaire. Only about half of the respondents visiting the website completed the questionnaire suggesting further selection. Due to data protection issues, we could only collect data of those completing the questionnaire and submitting the data. Therefore, we can only speculate on reasons for non-completion. Furthermore, a high proportion of participants had further qualifications or reported recent training in delirium. Hence, the results from our survey may overestimate delirium competence of nurses in Germany.

## Conclusion

The newly developed questionnaire was feasible and well-appreciated by respondents. The results of this study suggest that the overall recognition of delirium by nurses should be improved. The questionnaire could augment existing training activities in the future. Although not addressed, our results implicated a particular need for nurses in long-term care facilities to strengthen their delirium competence. This should be addressed in further research with an appropriate sample size. The authors would welcome use of case vignettes and access to the online questionnaire by German instructors.

## Supplementary Information

**Additional file 1.**

## Data Availability

The questionnaire was translated into English and can be seen in the supplementary data. The datasets used and/or analyzed during the current study are available from the corresponding author upon request.
