# Supplementary material for: Nurses’ competence in recognition and management of delirium in older patients: development and piloting of a self-assessment tool
Source: BMC Geriatr. 2022 Nov 19;22:879. doi: 10.1186/s12877-022-03573-8 (PMC9675220; doi:10.1186/s12877-022-03573-8)
Supplement: Supplementary file 1 — Additional file 1. [file 12877_2022_3573_MOESM1_ESM.pdf]

SUPPLEMENTARY MATERIAL

Case vignettes (English translation)

Vignette No. 1

An 84-year-old man was admitted to hospital due to an infection of a venous ulcer on the right lower leg. He was accompanied by his wife. While taking the nursing history, the patient presented fully oriented and gave a spry and talkative impression. He reported increased pain in the leg with a NRS (pain scale of 0 to 10) of 6. Other values included blood pressure (RR) of 140/80 mmHg, pulse of 104 bpm, and blood glucose (BG) of 252 mg/dl with a known type 2 diabetes mellitus. The wife reported that they had been managing well at home and only needed assistance with wound care of the ulcer. The patient had been mobile with a walking stick, but had recently lost some mobility as is typical for old age. During the admission day, the patient underwent several tests outside the ward. In addition, the pain medication was adjusted and antibiotics were started. Paracetamol and metamizole were prescribed as on-demand medication.

The next afternoon, during handover colleagues of the morning shift report that the patient had appeared agitated and anxious during the previous evening and night, but had slept "well" after a single dose of on-demand medication (diazepam 5mg). In the morning he had been inconspicuous. Later that afternoon, the wife calls the ward and reports excitedly that her husband had just called her and talked about his immediate discharge. You then go to see the situation for yourself and find the patient upset and packing his suitcase. When asked, he states that his vacation is now over and that he has to go to work. During the conversation, the patient is uncooperative, insists on being discharged and continues to tamper with his suitcase.

Questions:

Are there any clues for you as to whether the patient described has acute and/or fluctuating alterations of state with respect to attention, consciousness, and/or thinking?

Your initial assessment: Which of the following conditions do you think could most likely apply to the situation described?

What measures should you still initiate or suggest in the current shift to prevent the situation from getting worse?

- Avoid further administration of benzodiazepines, consult with medical staff for alternatives.

- Regularly review pain status and max out pain medication.
- Place a permanent bladder catheter to better monitor fluid balance.
- Provide a familiar and quiet environment for the patient at night, do not transfer him.
- Transfer the patient to the monitoring ward for better observation of vital signs.
- Give benzodiazepines (e.g., diazepam) for agitation, anxiety, and sleep problems.
- Attach bed rails after consulting with wife.
- Ask the wife to come to the hospital to de-escalate the current situation.
- Discuss the situation with the wife, then lock away suitcase and walking stick so the patient cannot leave the ward.
- None of the above measures are required.

Vignette No. 2

A 79-year-old man was admitted to the surgical ward with femoral fracture after a domestic fall. The fracture was immediately treated surgically. The patient's nursing history was taken postoperatively mainly from the patient’s wife, as the patient had been repeatedly "somewhat forgetful" in recent years. Due to the forgetfulness in everyday life, he needed support in personal hygiene and assistance with more complex activities of daily living. The wife stated that she would care for her husband independently at home. He has level of care of 3 [out of 5], and she would be able to manage his care well. Prior to the fall, the patient had been mobile with his walker inside the house as well as the garden according to the wife. During the further course of treatment, the patient repeatedly complained of pain and was visibly unhappy about his immobility. For this reason, he had been given tramadol as an on-demand medication on one occasion. Nursing staff reported increasing apathy and sleepiness during the day and insomnia at night. However, when this is discussed during the ward round the patient appeared awake and responsive, and his expressions seemed adequate.

As the day progresses, the patient is found sleeping again, tablets and lunch untouched. You wake him up and prepare his lunch. However, you notice, that food intake is unusually difficult. The patient tries to spoon the soup with a fork and stops eating after a short time. You encourage him again, but the patient cannot focus on what you are saying and after a short time his eyes already fall shut. The wife is visibly surprised when she arrives for her daily visit, as her husband is "a good eater" and she has never had to support him in this. In addition, he seems much more apathetic than usual, so that she

would hardly recognize him. During the conversation, the patient does not seem to fully comprehend and has difficulties following the conversation.

Questions:

Are there any clues for you as to whether the patient described has acute and/or fluctuating alterations of state with respect to attention, consciousness, and/or thinking?

Your initial assessment: Which of the following conditions do you think could most likely apply to the situation described?

You give the newly assigned ward physician a brief assessment of the patient during the ward round. What statements would you make about the patient's mental status?

Q1.

- The wife's description suggests dementia.
- The wife reports age-appropriate forgetfulness.
- There is no evidence of a cognitive problem from the wife's description.

Q2.

- Postoperatively, increased fatigue and decreased drive are noticeable.
- Postoperatively, the patient is still very exhausted and therefore sleeps a lot.
- Postoperative behavior is unremarkable. Due to the new environment, the patient can currently sleep poorly at night.
- In the context of known dementia, the patient's day-night rhythm is disturbed and his attention is reduced.

Q3.

- The wife reports a change in personality. The symptoms are fluctuating.
- He is not very cooperative because he is close-knit to his wife. The wife appears overprotective.
- He is still exhausted from the anesthesia and therefore has little appetite.
- The unfamiliar food affects his appetite and he currently eats poorly. Due to dementia, he cooperates poorly.

Q4.

- There is a high probability of hypoactive delirium.

- The postoperative course is typical for patients with dementia.
- There is an accelerated progression of dementia.
- The postoperative course is unremarkable.

Q5.

- There is a need for action.
- The further course of events should be awaited for the time being.

Vignette No. 3

An 82-year-old man is cared for on a surgical ward because of a humerus fracture. After a fall from a ladder at home, he alerted the ambulance service independently. The patient lives alone and has been independent. During the nursing history, the daughter reported that during the course of the last year, the patient's wife had died of SARS-CoV2. Since then, the father had lived in seclusion in the house and left it only for shopping. He would also have drunk a bottle of beer and a glass of wine every day since the wife's death. The patient had not mentioned this in his medical history so far. Currently, he requires nursing assistance due to the fracture and has a Barthel index score of 70 out of 100. Due to the fall, he is very anxious and does not dare to walk alone on the corridor. When asked, he states that he has no pain, but reports permanent fatigue and listlessness. Measurements of vital signs reveal no abnormalities. During the hospital stay, it is observed that the patient continues to withdraw and appears unmotivated. The patient reports that he has problems falling asleep at night. Thought processes appear slowed to staff, but he appears fully oriented. Further care after discharge is to be discussed together with the patient's daughter.

Questions:

Are there any clues for you as to whether the patient described has acute and/or fluctuating alterations of state with respect to attention, consciousness, and/or thinking?

Your initial assessment: Which of the following conditions do you think could most likely apply to the situation described?

You would like to take appropriate measures to prevent delirium. What are the most suitable measures for the 82-year-old man in the case described?

- In consultation, suggest psychiatric consultation for assessment and treatment of possible depression.
- Use benzodiazepines to prevent possible withdrawal symptoms.
- Place indwelling catheter because of patient's fear of falling and spending most time in bed.
- Attach bed rails for fall prevention.
- Consult with physician to increase pain medication.
- Discussion possible alcohol abuse during the ward round and initiate further measures, if necessary.
- Create or actively provide orientation (clock, calendar, daily newspaper) and employment opportunities.
- Discuss all further decision-making processes with the daughter and no longer involve the patient in order to relieve the latter.
- Provide support and guidance with mobilization to reduce fear of falling.
- No action is required.

Vignette No. 4

An 88-year-old woman cared for on a ward with pelvic fracture. She has been cared for at home by her daughter, who lives in the same house, after the death of her husband. The patient needs assistance with all activities of daily living (ATLs). According to records from the family physician, the patient has congestive heart failure, osteoarthritis, and asthma. About five years ago, the patient had suffered a stroke; Mini Mental State Examination at that time was 24 out of 30. Since the daughter had wanted to go on vacation, she had registered for respite care and organized a short-term care facility for her mother. During this stay, the patient fell from her bed while getting up and had to be admitted to hospital as an emergency case. On the ward, the patient complains of severe pain but does not independently ask for on-demand medication. This is done by her neighboring fellow patient, who seems visibly stressed, because the woman keeps calling for her. The patient does not leave her bed independently. In conversation, the patient repeatedly asks both medical staff and nursing staff about her current whereabouts as well as her husband, and she seems to have little orientation. Furthermore, word-finding difficulties are noticeable. The daughter cannot be reached for clarification of further therapy; required documents are brought by the grandchildren. The grandchildren are not involved in the patient's care, but would not describe much

change of their grandmother’s general appearance. They report that their grandmother had become "forgetful as is typical for old age” in recent years.

Questions:

Are there any clues for you as to whether the patient described has acute and/or fluctuating alterations of state with respect to attention, consciousness, and/or thinking?

Your initial assessment: Which of the following conditions do you think could most likely apply to the situation described?

Through consultation with the family doctor and in the context of a discussion in the ward team, you come to the conclusion that a dementia development is probably present. This should be tested separately in the course to obtain a confirmed diagnosis. Currently, however, there seem to be no acute changes in the cognitive state. Since age and an existing cognitive deficit are potential risk factors for delirium, you would like to take further preventive measures. What are the most suitable measures for the 88-year-old patient?

- Perform room changes with the patient so that the other co-patient is relieved.
- Use of benzodiazepines or haloperidol to reduce calling.
- Provide daily and multiple physical and cognitive activation, e.g., by taking meals while seated.
- Attach bed rails for fall prevention.
- Ask medical staff to increase regular pain medication.
- Remind patient to be quiet and point out the patient bell.
- Create temporal orientation through calendar and clock.
- To reduce anxiety, have relatives bring familiar objects and pictures.
- Put notes at the bedside to remind the patient that her husband has already died.
- Place indwelling catheter to compensate for present incontinence and currently reduced mobility.

Vignette No. 5

A week ago, a 73-year-old woman was admitted to the emergency department with a febrile infection and reduced general condition. There, in addition to the infection, the diagnosis of acute leukemia was made. Thereupon, the woman was transferred to the hematology ward. In addition to further extensive diagnostic tests, oral chemotherapy

was started immediately. In addition, the patient received antibiotics four times a day and 2 liters of saline intravenously daily. Due to the almost continuous intravenous injections, she is limited in her mobility. During the first week, the patient is perceived by the ward team as combative and highly motivated to overcome the disease. Her goal is to regain full independence and to return to her home. She comes across to the team as well-informed and requiring only occasional nursing assistance.

After a week on the ward, the woman is found unusually sleepy during your morning nursing round. Moreover, breakfast and the morning medication are not touched. When asked what is going on today, the patient only makes brief eye contact and answers with a time delay and she speaks with a slur. After some "time to wake up" and care for other patients, the patient is suddenly found awake and eating. She appears slowed down, is not oriented to time when asked, and believes she is eating dinner. She negates pain, nausea or other problems.

Questions:

Are there any clues for you as to whether the patient described has acute and/or fluctuating alterations of state with respect to attention, consciousness, and/or thinking?

Your initial assessment: Which of the following conditions do you think could most likely apply to the situation described?

Which assessment tool do you consider suitable for delirium clarification in your everyday professional life? (Multiple answers possible)

Figure: Characteristics of the case vignettes and scoring of the GDCA

Clinical case situations:

Case 1: hyperactive delirium	Case 2: hyperactive delirium superimposed on dementia	Case 3: hypoactive delirium	Case 4: depression	Case 5: dementia

Characteristics and recognition (clinical judgment):

- each case: signs of acute onset or fluctuating course of mental status(yes/no) 1 point / vignette
- each case: delirium presence (correct / false) 1 point / vignette
- each case: diagnoses (one diagnosis out of 15) 1 point / vignette

Management tasks (clinical decision making):

Management tasks were composed of multiple-choice questions with several correct options. In case 1,4 and 5 participants had to select measures from a catalog. No information about the number of correct choices were given. In case 2 a dialogue had to be completed.

Case 1: treatment of delirium up to 10 points
Case 2: interdisciplinary communication up to 10 points
Case 4: recognition of risk factors and initiate preventive measures up to 10 points
Case 5: prevention for patients with cognitive impairment up to 10 points

Overall:
Total
55 points

Recognition:
Total
15 points

Management:
Total
40 points
